# Educational Intervention for Management of Acute Trauma Pain: A Proof-of-Concept Study in Post-surgical Trauma Patients

**DOI:** 10.3389/fpsyt.2022.853745

**Published:** 2022-07-04

**Authors:** Luana Colloca, Ariana Taj, Rachel Massalee, Nathaniel R. Haycock, Robert Scott Murray, Yang Wang, Eric McDaniel, Thomas M. Scalea, Yvette Fouche-Weber, Sarah Murthi

**Affiliations:** ^1^Department of Pain and Translational Symptom Science, School of Nursing, University of Maryland, Baltimore, Baltimore, MD, United States; ^2^Department of Anesthesiology, School of Medicine, University of Maryland, Baltimore, Baltimore, MD, United States; ^3^Medical Training Program, School of Medicine, University of Maryland, Baltimore, Baltimore, MD, United States; ^4^R Adams Cowley Shock Trauma Center, University of Maryland, Baltimore, Baltimore, MD, United States; ^5^Center to Advance Chronic Pain Research, University of Maryland, Baltimore, Baltimore, MD, United States

**Keywords:** opioids, surveys, education, expectations, perceptions, trauma-related pain, post-operative monitoring

## Abstract

**Objective:**

Despite years of research and the development of countless awareness campaigns, the number of deaths related to prescription opioid overdose is steadily rising. Often, naive patients undergoing trauma-related surgery are dispensed opioids while in the hospital, resulting in an escalation to long-term opioid misuses. We explored the impact of an educational intervention to modify perceptions of opioid needs at the bedside of trauma inpatients in post-surgery pain management.

**Materials and Methods:**

Twenty-eight inpatients with acute post-surgical pain completed this proof-of-concept study adopting an educational intervention related to opioids and non-pharmacological strategies in the context of acute post-surgical pain. An education assessment survey was developed to measure pre- and post-education perceptions of opioid needs to manage pain. The survey statements encompassed the patient’s perceived needs for opioids and other pharmacological and non-pharmacological therapeutics to manage acute pain. The primary outcome was the change in the patient’s perceived need for opioids. The secondary (explorative) outcome was the change in Morphine Milligram Equivalents (MME) used on the day of the educational intervention while inpatients and prescribed at the time of the hospital discharge.

**Results:**

After the educational intervention, patients reported less agreement with the statement, “I think a short course of opioids (less than 5 days) is safe.” Moreover, less agreement on using opioids to manage trauma-related pain was positively associated with a significant reduction in opioids prescribed at discharge after the educational intervention. The educational intervention might have effectively helped to cope with acute trauma-related pain while adjusting potential unrealistic expectancies about pain management and, more in general, opioid-related needs.

**Conclusion:**

These findings suggest that trauma patients’ expectations and understanding of the risks associated with the long-term use of opioids can be modified by a short educational intervention delivered by health providers during the hospitalization. Establishing realistic expectations in managing acute traumatic pain may empower patients with the necessary knowledge to minimize the potential of continuous long-term opioid use, opioid misuse, and the development of post-trauma opioid abuse and/or addiction.

## Introduction

According to the Centers for Disease Control and Prevention (CDC), an average of 38 deaths occurred *each day* in 2019 involved opioid prescriptions in the United States (1). The CDC newly released data reported an increased estimation of overdose deaths over the last year (time period ending in April 2021) with 75,673 from the previous year 56,064 overdose deaths (2). One out of 550 chronic opioid users dies approximately within 2.5 years of their *first* opioid prescription to treat acute pain, meaning that many opioid-related deaths can be prevented by addressing them within the acute care setting (3).

Physically injured trauma patients are challenging to follow long-term, and as a result, information on the precise number of patients that develop opioid dependence and potential death due to prescription opioids for acute traumatic pain remains unknown. However, in a recent study of 36,000 opioid-naive patients undergoing elective surgery, continuous opioid use was 6% regardless of whether the surgery was minor or major (4). The R Adams Cowley Shock Trauma Center at the University of Maryland, Baltimore admits about 8,000 inpatients each year from the State of Maryland, the vast majority of whom are prescribed opioids during their admission (5). Assuming 85% of inpatients are given an opioid (5) and assuming an estimated 6% rate of long-term opioid use (4), at least 400 patients each year could use opioids continuously with the potential to develop opioid dependence due to initial opioid-based treatment of trauma injuries. Moreover, trauma patients identified as at-risk drinkers at the time of injury were found to have a 7–10% rate of non-medical use of prescription opioids at 1 year (6). Injured trauma patients with severe pain are hospitalized for long periods and given high doses of opioids throughout their hospitalization. Disjointed medical care between inpatient and outpatient settings often results in little to no continuity of pain management, and potentially, no adequate plan to taper and stop opioid use. Factors that may put patients at risk of developing opioid misuse and dependence include the patient expectation of being “pain-free” despite significant injuries, as well as a lack of awareness regarding the dangers of long-term opioid use at the time of initiation (7–10). Therefore, we designed and conducted a pilot study to test the effects of an educational intervention on expectations of opioids’ needs and quantifiable outcomes (pain, opioid use) in trauma inpatients. Our main question was: Can we optimize patients’ expectations of opioids’ needs to optimize the risks/benefits of opioids used for trauma-related pain? Expectations are predictions of future outcome(s) based on pre-existing individual assumptions constructed from current personal knowledge and previous experiences (11). Expectations about the effectiveness of the treatment can influence patient outcomes, drug intake and behaviors (12, 13). A recent study conducted during the pre-surgery window of patients undergoing heart surgery, demonstrated that an educational session targeting expectations related to the post-surgery recover and outcomes as compared to standard information, improved post-surgical heart-related outcomes including lower post-surgery interleukin-6 level, mental health and hours of work per week at 6 months from the surgery (14).

It is well documented that long-term use of opioids for non-cancer pain treatment resulted in opioid misuse and poor pain management (15). In the attempt to reduce opioid misuse and addiction, an effort has been made on reducing patients’ needs for opioids after surgery (7–9) but there was a paucity of studies focusing on educational interventions related to tapering acute opioid intake for patients with traumatic injuries. Based on this knowledge, this proof-of-concept study intended to understand whether expectations of opioid needs in post-trauma inpatients can be modified by using an educational intervention to reduce the perception of opioids’ needs (primary outcome). Changes in Morphine Milligram Equivalents (MME) used on the day of the educational intervention while inpatients and prescribed at the hospital discharge were also collected (explorative outcomes).

## Materials and Methods

Thirty-one trauma inpatients were enrolled in this study to test the impact of an educational intervention on the patient’s understanding of the need for opioids and expectancies about opioid needs to manage pain. We had three dropouts. Three inpatients were unable to complete the study due to ongoing clinical procedures, physical therapy and sleeping, leaving a total sample of 28 patients with complete data (20 women and 8 men). Sociodemographic and clinical characteristics, including age, sex, race, educational status, and marital status, were collected at the baseline before the educational intervention.

The study took place at the R Adams Cowley Shock Trauma Center at the University of Maryland Medical Center from April 2019 to February 2020. The study required about 1 h and inpatients hospitalized for trauma were invited to participate by research staff independent of the health clinicians treating patients. All patients provided their verbal and written informed consent to participate in this study. Participants were made aware that they had the right not to participate and withdraw from the study. Participation was entirely voluntary with no monetary incentive. The University of Maryland R Adams Cowley Shock Trauma Center and the University of Maryland Institutional Review Board approved this study (HP-00083434) and all procedures were conducted in accordance with the Declaration of Helsinki ethical principles for medical research involving human beings.

### Inclusion/Exclusion Criteria and Enrollment

The research coordinators accessed medical records from the hospital clinical database ‘EPIC’ to identify potential trauma inpatients who met the inclusion criteria for enrollment. For this study, we considered trauma as a severe but not life-threatening single or multiple injuries that had required immediate medical treatment to help treat the trauma at our R Adams Cowley Shock Trauma Center.

Eligible inpatients were admitted to the University of Maryland R Adams Cowley Shock Trauma Center for trauma-related incidents. These inpatients had to be between the ages of 18–65 years. The trauma related incidents could have been related to several injuries from motor vehicle crash injuries, blunt-force, stab and gunshot-induced wounds, falls resulting in single and/or multiple broken bones. The patients were also eligible if they received opioids for acute pain, and were opioid naïve before being admitted to the emergency rooms (i.e., had not been treated with opioids daily for the past 3 months).

The exclusion criteria included a lack of English fluency, inability to sign informed consent, cognitive impairment, illiteracy, diagnosis of diffuse cancer (excluding those that are isolated or benign tumors that do not require treatment), or a planned enrollment into a substance abuse treatment program that prescribed medications (e.g., Suboxone or Methadone), and trauma related to major head concussions or other injuries. We excluded those taking opioids for cancer pain and for drug addiction/abuse disorders to target opioid-naïve patients. The long-term goal is to limit the escalation of the opioid epidemic, particularly for those who are initially exposed to opioids because of traumatic injuries.

**FIGURE 1:**
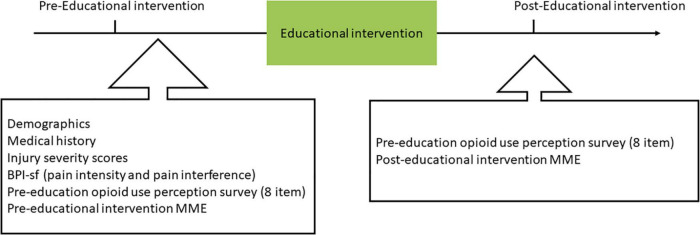
Study procedure. Inpatients’ demographics, medical history, opioid intake, trauma injury severity, clinical pain intensity and interference, and opioid use perception were obtained before the educational intervention. After the educational intervention, the opioid use perception was re-assessed. Opioid prescription expressed in Morphine milligram equivalents (MME) were measured at discharge based on the medical records.

Once potential inpatients were identified, one of the research coordinators scheduled a time with the team of nurses and doctors to approach the patient at the bedside and invite him/her to participate in the study (see Figure 1 for study time line). It was made clear to inpatients that there were no benefits in participating in the study, participation was entirely voluntary, and the decision to partake in the study or not had no impact on the course of the medical treatment. Inpatients were asked whether they were interested in participating in a study to learn more about pain management options, including opioids and non-opioids and their related risks.

### Educational Intervention

**FIGURE 2:**
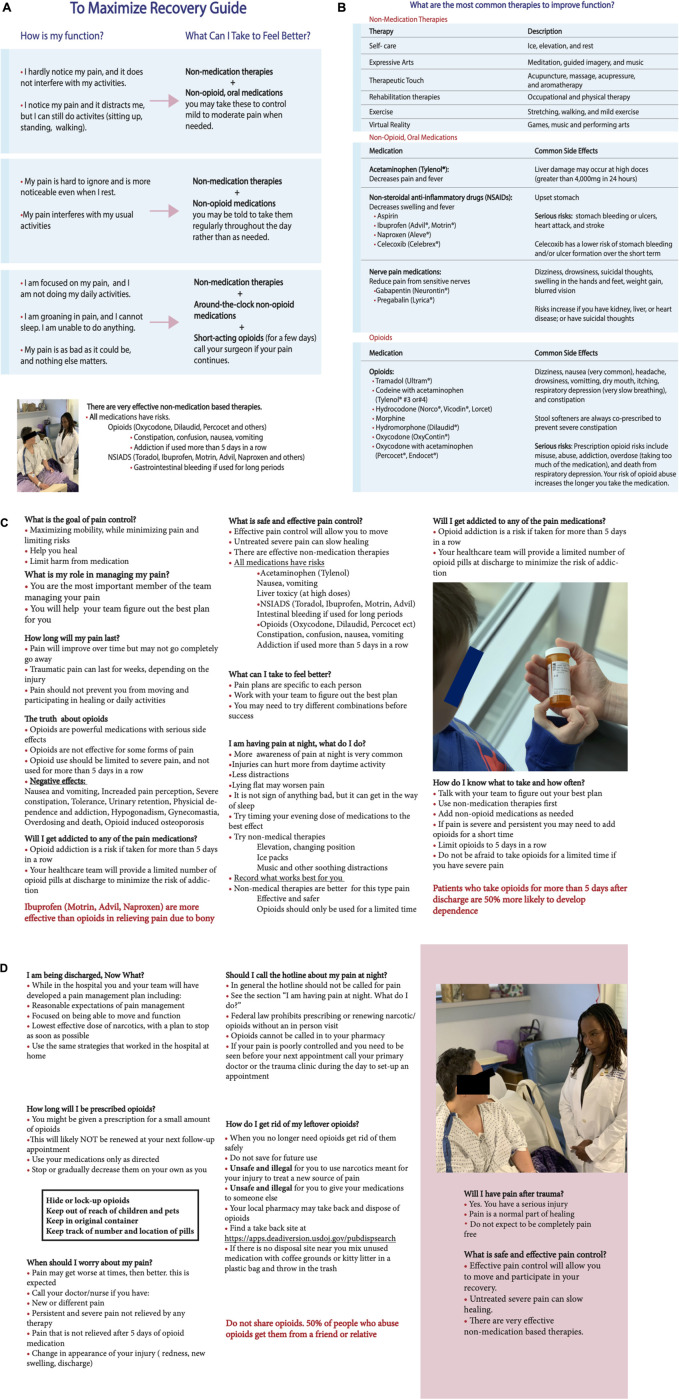
Educational intervention brochure. It started with a recovery guide that aimed to maximize patient recovery by explaining the pain interference (“How is my function?”) and the pharmacological and non-pharmacological therapeutic options (“What can I take to feel better?”) **(A)**. The educational brochure provided then a list of the most common therapies to improve functions **(B)**. The remainder of the educational materials had commonly asked questions and answers to topics specific to pain control goals, duration of pain, and risks of addiction development **(C,D)**.

The materials for the educational intervention were developed by LC, YF-W, and SM. They engaged in several focus group sessions with the nurses and doctors from the R Adams Cowley Shock Trauma Center at the University of Maryland Medical Center, receiving feedback in preparing the brochure and related survey content. Feedback on the educational intervention goals and endpoints was also received by the Center for Addiction Research, Education, and Service (CARES), University of Maryland, Baltimore. The educational intervention consisted of a brochure containing modified parts of the Surgical Patient Education Program developed by the American College of Surgeons (16) and other parts tailored to the Shock Trauma Center context. The educational intervention started with a recovery guide that aimed to maximize patient recovery by explaining the pain interference (“How is my function?”) and the pharmacological and non-pharmacological therapeutic options (“What can I take to feel better?”) (see Figure 2A). The educational brochure provided a list of standard therapies to improve pain and functions (Figure 2B). For example, options for non-medication therapies and their descriptions were listed to provide alternatives to pain management. These non-medical options included self-care, expressive arts, therapeutic touch, rehabilitation therapies, exercise, and the use of virtual reality. Patients were given additional information classifying medications as either non-opioids or opioids and listing the common side effects for each medication. The remaining educational materials contained commonly asked questions and answers to topics specific to pain control goals, duration of pain, and risks of addiction development (Figures 2C,D). Consideration about both short-term and long-term opioid misuses was also provided to inpatients as part of the educational intervention. Information pertaining to this study was delivered by the research coordinators verbally and in the form of an educational brochure. Treating doctors and nurses were not involved in the study to avoid recruitment biases and risks of patients’ coercion.

### Pre- and Post-education Assessment Survey

In order to assess the effect of the educational intervention, a survey was developed to record pre- and post- perceptions toward opioids and non-opioids use. The research team developed the survey questions on the research hypotheses. The same education assessment survey was given before and following the educational intervention. In the survey, inpatients rated their levels of agreement to eight statements by marking along a 10 cm horizontal scale. The participant markings were measured in centimeters and ranged from “definitely no” (0 cm) to “definitely yes” (10 cm). Following the intervention, the same survey was given again to inpatients, but they were asked two additional questions regarding their perceived utility of the educational intervention and how easy it was to understand the presented educational materials. Responses to these two items were also measured along with the horizontal agreement visual analog scale. The education assessment survey tool is presented in Supplementary Table 1.

### Brief Pain Inventory Short Form

Prior to filling out the surveys and undergoing the educational intervention, inpatients were asked to fill out a Brief Pain Inventory Short Form (BPI-SF) (17) to measure the pain they were experiencing before the intervention. Inpatients were asked to rate their worst, least, average pain during the past 24 h and current pain by circling a discrete number from 0 to 10, with 0 being “no pain” and 10 being “pain as bad as you can imagine.” The average score of the 4 items were used to represent pre-educational pain intensity.

### Injury Severity Score

Injury Severity Scores (ISS) were calculated for all inpatients enrolled. Scores were calculated by assigning an Abbreviated Injury Scale (AIS) code for each injury of each body region. The ISS score is then calculated by adding the sum of the squares of the 3 highest codes in the 3 most injured body areas (18; 19).

### Morphine Milligram Equivalents – Explorative Outcome

Opioids were administered via the oral and intravenous routes. Opioid intake and prescriptions were calculated in MME for each participant by the sum of each prescribed opioid medication multiplied by its respective conversion factor (20). The MME were calculated at the time of the educational intervention and post-education at hospital discharge.

### Statistical Analyses

Sociodemographic and clinical characteristics are reported as percentages to examine how perception toward opioids could have changed before and after the educational intervention, calculated by ANCOVA controlling for age, sex, and race. We used agreement ratings to each of the statements assessed pre- and post-educational intervention. In particular, the two time-points (pre- vs. post-educational intervention) were set as a within-subjects factor while age, sex, and race were treated as covariates.

In addition to the perception changes, we examined how educational intervention would have changed the dispensed opioids while inpatients and at the prescribed opioids at the hospital discharge. Therefore, MME on the day of educational intervention, and MME at discharge were calculated for each patient controlling for level of trauma severity (ISS scores) and pain severity (BPI-SF scores). ANCOVA was used to compare administered at the time of the educational intervention and prescribed MME at discharge controlling for level of trauma severity using ISS scores and pain severity using BPI-SF scores along with demographic variables age, sex, and race. Given that some medications were prescribed as ranges at discharge, we compared the minimal prescribed MME at discharge with the MME dispensed at the time of the educational intervention.

We used Spearman correlations to examine whether the changes in the attitudes toward opioids were correlated with the changes in prescribed opioids expressed as minimal dose of MME at discharge.

For the primary outcome (changes in attitudes toward opioids), a conservative Bonferroni corrected/adjusted *p*-value of 0.0062 dividing the 0.05 (α-value) by 8 which corresponds to total analyses on the dependent variable, was used for significance. On the contrary, an unadjusted *p*-value of 0.05 was used for the secondary explorative outcomes (dispensed MME and prescribed MME at the hospital discharge). SPSS statistics version 26.0 was used for all data analyses.

## Results

### Socio-Demographic and Clinical Characteristics

A total of 28 trauma patients completed the study. Eight out of 28 participants were men (28.57%) and 20 were women (71.43%). The average age of this cohort was 42.04 years with a 95% CI of 35.92–48.15 years. In terms of race, 13 out of 28 were White (46.42%), while the remaining 15 were African American/Black inpatients (53.58%). The majority of the cohort was non-Hispanic (25 out of 28, 89.29%) with the remaining three participants reporting unknown ethnicity. Regarding the socioeconomic status, most inpatients were never married (42.86%) with 39.29% of the cohort reporting a married or living as married status. Seven out of 28 inpatients had a college graduation or higher education (25%), while 20 out of 28 (71.43%) had some college or less education.

In terms of clinical factors, an ISS range of 16–24 indicates the presence of severe injuries, and the cohort had an ISS average of 19.96 out of 75, suggesting presence of severe injuries (Supplementary Table 2).

Type of injuries included degloving injury of lower limbs, single or multiple fractures of the ribs, femur (closed displaced sub-trochanteric part), proximal humerus (closed 3-part fracture), radius, tibia, fibula, calcaneus, vertebra and pelvis, laceration to spleen, laceration of the tongue, aortic arch pseudoaneurysm, pulmonary contusion, hemopneumothorax, diaphragm injury, empyema lung assault with gunshot wounds and stab wounds. In addition, baseline pain intensity levels had an average of 6.26 out of 10 pain intensity ratings, indicating moderate to severe pain intensity levels for this trauma inpatient cohort.

### Attitudes Toward Medical and Non-medical Treatments Pre- and Post-educational Intervention

As expected, before the educational intervention, inpatients had medium levels of agreement with the statement “I think all pain related to my trauma is bad and should be treated” with a mean of 6.85 (SEM = 0.79) out of a total rating of 10 (from 0 = definitely disagree to 10 = definitely agree). This attitude did not significantly change after the education as revealed by the non-significant ANCOVA controlling for age, sex, and race (post-education: mean = 6.18, SEM = 0.71, *F*_1,24_ = 0.36, *p* = 0.555). Similarly, inpatients’ attitudes toward personal involvement, “I think I am an important part of the team treating my pain,” did not significantly change after the educational intervention (*F*_1,23_ = 0.002, *p* = 0.964).

Attitudes toward the importance of using opioids/narcotics for trauma-related acute pain (*F*_1,24_ = 1.12, *p* = 0.301) and the timeframe of using opioids (*F*_1,24_ = 0.41, *p* = 0.528) for pain management did not significantly change as a result of the educational intervention.

**FIGURE 3:**
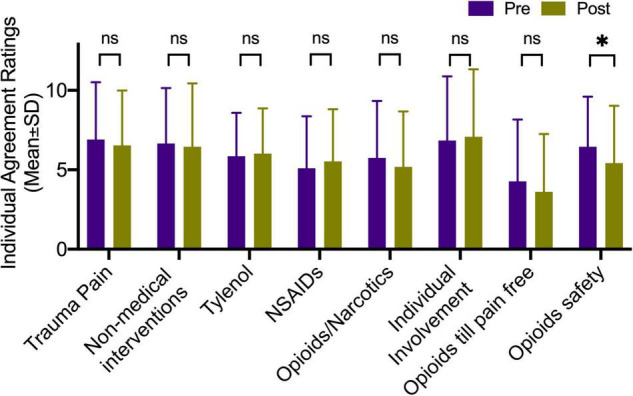
Educational intervention changes. Degrees of agreement to the statements regarding the importance of pharmacological and non-pharmacological treatments for acute trauma pain. Opioids safety significantly changed with a decreased mean score of 6.92 (SEM = 0.60) at the pre-education phase as compared with 6.09 (SEM = 0.72) at the post-educational intervention (*F*_1,24_ = 9.08, *p* = 0.006) controlling for age, sex, and race. *Indicate statistical significance.

Notably, the answer to the statement “I think a short course of opioids (less than 5 days) is safe” significantly changed with a decreased mean score of 6.92 (SEM = 0.60) at the pre-education phase as compared with 6.09 (SEM = 0.72) at the post-educational intervention (*F*_1,24_ = 9.08, *p* = 0.006 with an adjusted value of *p* = 0.048) controlling for age, sex, and race (Figure 3). This suggested the educational intervention significantly changed, specifically the beliefs about the safety of opioids. The attitudes toward acetaminophen such as Tylenol (*F*_1,22_ = 2.81, *p* = 0.108) and NSAIDs such as Advil or Motrin (*F*_1,22_ = 2.55, *p* = 0.125) did not significantly change after the educational intervention when compared to the pre-education phase.

In terms of the attitude toward the use of non-pharmacological interventions such as music and meditation, there were no significant changes for the statement “*I think non-medical interventions are an important part of my treatment plan*” after the educational intervention controlling for age, sex, and race (pre-education: mean = 6.64, SEM = 0.72; post-education: mean = 6.44, SEM = 0.82, *F*_1,24_ = 0.02, *p* = 0.883).

### Prescribed Opioids and Educational Intervention

**FIGURE 4:**
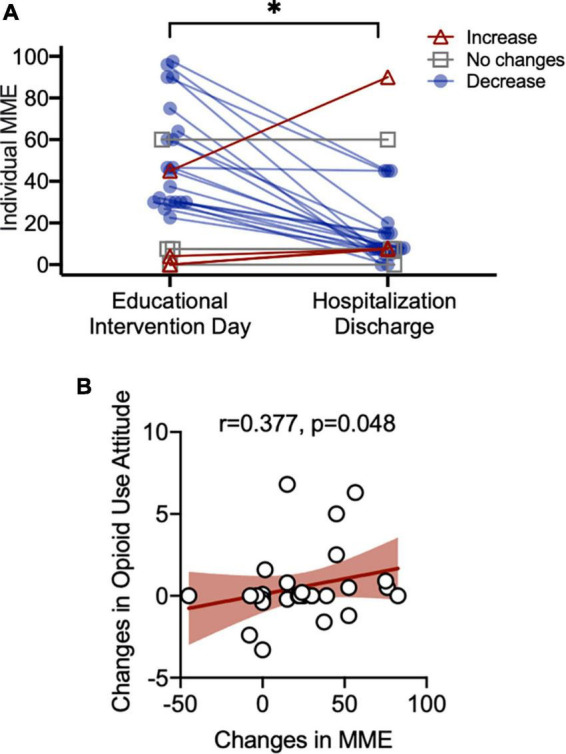
Morphine milligram equivalents (MME) changes **(A)**. Controlling for age, sex, and race, post-education opioid prescription (minimal amount) were significantly lower than the pre-education actual opioid intake (*F*_1,24_ = 4.53, unadjusted *p* = 0.044). Attitudes toward opioids and MME reductions **(B)**. Spearman correlation between reduction in attitudes toward opioids safety/needs and MME reductions (Spearman *r* = 0.377, unadjusted *p* = 0.048). *Indicate statistical significance.

The prescribed opioids were calculated as MME on the day of the educational intervention and post-education at the day of discharge. ANCOVA indicated that the opioid intake significantly dropped from an average of 42.26 MME (SEM = 5.55) per day, to an average of 16.98 (SEM = 4.08) prescribed MME per day (*F*_1,21_ = 4.47, unadjusted *p* = 0.047) while controlling for trauma and pain severity along with age, sex, and race. The majority of the inpatients (*n* = 20) experienced a reduction in opioids intake during the post-education phase compared to the day of education, controlling for severity of trauma and clinical pain. Only four inpatients had a higher opioid intake after the educational intervention and four other inpatients remained at the exact dosage of opioid intake from the day of the education to the post-education phase (Figure 4A).

Next, we examined whether reductions in opioid intake were related to the changes in the attitudes toward the use of opioids for management of acute trauma pain. Spearman correlations indicated a significant positive correlation between the reductions in overall opioids expressed in MME and attitudes toward the importance of considering opioid safety even as a treatment for acute trauma-related pain (spearman *r* = 0.377, unadjusted *p* = 0.048, Figure 4B). This result suggested that inpatients who changed their thoughts about the safety of opioids in acute pain management after the educational intervention had greater reductions in prescribed MME opioids at discharge, post-educational intervention.

### Patients’ Feedback

When we asked whether being informed was helpful, 19 out of 28 inpatients reported and found the educational intervention useful, helpful, and easy to understand. This was demonstrated by an agreement score of 9.21 ± 1.41 out of 10 to the statement “I think the information shown to me was useful/I could use it to help manage my traumatic pain” and an agreement score of 9.71 ± 0.46 out of 10 to the statement “I think the information shown to me was easy to understand.” The rest of the inpatients did not indicate whether the information shown was useful, helpful, or easy to understand.

## Discussion

The current study aimed to educate trauma patients about the potential danger of opioid usage while introducing alternative methods to improve acute pain management. Our goal was to demonstrate with this proof-of-concept study that the educational intervention could improve the patient’s understanding of the need for opioids. We found that a short educational intervention could shift attitudes positively toward concerns about the safety of short-term opioids. The educational intervention did not change the perception of using other medical treatments (e.g., acetaminophen, NSAIDs) and non-medical approaches (e.g., music, meditation). More importantly, larger reductions in the perception of opioid safety were correlated with greater reductions in the prescription of MME at discharge, hinting to a potential relevance of education as an adjuvant intervention for pain management.

A traumatic injury can be a gateway to chronic opioid misuse (10). In the current study, we provided trauma patients with educational interventions by giving verbal and written information about the safety and effectiveness of post-trauma pain therapeutics. The educational intervention aimed to convey the concept that the ultimate goal of post-trauma pain control was not merely to achieve a pain-free status. Rather, the purpose of the acute pain management plan was to facilitate the healing process and maintain daily activities while minimizing pain interference. Stopping both short-term and long-term opioid misuses was the focus of our proof-of-concept study involving the development of an *ad hoc* created educational intervention. As hypothesized, after the educational intervention, we observed reductions in prescribed opioids directly related to the shift in attitudes toward opioids. In addition, another recent pilot study employed video-based education on opioid safety for patients after traumatic injury (21). The authors found that among the patients who were continuously using opioids throughout the study, the group who watched the educational video had lower MME than the group who did not receive an educational information (21).

While the educational intervention did not influence attitudes toward NSAIDs, or complementary and alternative pain interventions, the educational intervention adjusted the perception of opioid usage by decreasing the positive attitudes toward the safety of short-term usage of opioids. In an explorative manner, we found that the adjustment in the perception of using opioids was associated with the changes in prescribed opioids. This finding echoed a previous study conducted in cancer patients where negative beliefs about opioids were associated with worse opioid adherence (22). In fact, expectations about treatment effectiveness (23, 24) and treatment beliefs (25) can influence clinical outcomes, including medication use and disease-related behaviors. In acute pain management, pain relief expectations have contributed to less clinical pain experiences via placebo mechanisms (23). More evidence found in a previous study showed that preoperative patient education effectively reduces post-operative narcotic pill consumption by changing expectations that modulates symptom perceptions and prescription needs (26). An individual may be less likely to seek higher dosages, additional opioid medications, or continuous usage if they understand that their opioid medication is not intended to resolve (zero out) all their pain, nor is the duration of their prescription dependent on the presence of pain (27). It should be noted that trauma patients in the current study held relatively positive attitudes to pharmacological treatment and non-pharmacological interventions such as music and meditation at both the pre-educational and post-educational phases, suggesting that patients were open to a variety of pain management therapeutics.

### Strengths and Limitations

There are some strengths related to this study. This study adopted an educational intervention to change expectations and perceptions toward opioids safety. Unlike a recent study using pre-recorded video education with a low participant compliance (21), the educational intervention in the current study was conducted by trained research staff, ensuring interactive educational procedures rather than passive learning. Moreover, the research staff was independent of the clinical treating team of nurses and physicians, minimizing recruitment and other biases. Notably, the independent research investigation ensured that clinical care was delivered without interfering with the standard pain management.

Second, the current study quantified the changes of perception of opioids at time of the educational intervention while being inpatients and when the patients were discharged (e.g., prescribed MME). In the current study, we found that the changes in the perception of opioids needs/safety were significantly correlated with the reduction in prescribed opioids, suggesting beliefs or expectancies were optimized to improve medication consumption.

Despite the strengths of the current study, there were several limitations. First, the MME on the day of discharge was collected based on the EPIC medical records. Therefore, findings from the current study reflected only prescribed opioids instead of the actual opioid intake. Also, an assessment of dispensed pre-and post-intervention MME would have been optimal. However, coordinating research activities at the bedside with trauma inpatients is highly challenging (28). Second, this study adopted a cross-sectional within-subjects design where all inpatients received the educational intervention. Without a control group (i.e., treatment as usual), the reductions in dispensed and prescribed opioids observed in the current study could be a mix of post-educational effects and natural recovery. Also, trauma patients are heterogeneous with large variations in age, type of trauma, hospitalization stay and pain levels. As a proof-of-concept study, we enrolled a relatively small number of inpatients, and thus, these findings cannot be generalized to a larger population. Future studies with larger sample size and adequate control groups (e.g., no-education and/or natural history group) are needed to examine the effectiveness and efficacy of the educational intervention on three aspects: (1) attitudes toward the perception of opioids needs during the hospitalization and post-discharge recovery, (2) opioid intake during the hospitalization and at home, and (3) prescribed and actual long-term usage of MME for trauma patients using for example, ecological momentary assessments (29). Despite these limitations, these findings outline the potential advantage of introducing educational in-person or video-based interventions to help trauma patients navigate recovery and post-traumatic acute (and chronic) pain management.

## Conclusion

Overall, the verbal and written education toward acute pain management delivered as an adjuvant intervention can significantly convey that short-term use of opioids is not meant to zero-out pain and can present substantial risks related to long-term use of opioids and paucity of evidence realted to tapering strategies (30). Moreover, the amount of opioids prescribed at the time of discharge as compared to the day of education decreased and was directly related to the changes of opioid perception. Caution shall be applied in drawing definite conclusions due to the proof-of-concept nature of the study; these findings suggest that implementing educational interventions at the bedside could effectively help to cope with acute trauma-related pain while adjusting potential unrealistic patients’ expectancies about opioid needs. With education and monitoring, fewer incidences of opioid abuse and, perhaps, addiction would likely develop.

## Data Availability Statement

The original contributions presented in this study are included in the article/Supplementary Material, further inquiries can be directed to the corresponding author/s.

## Ethics Statement

The studies involving human participants were reviewed and approved by the University of Maryland Institutional Review Board approved this study (HP-00083434). The patients/participants provided their written informed consent to participate in this study. Written informed consent was obtained from the individual(s), and minor(s)’ legal guardian/next of kin, for the publication of any potentially identifiable images or data included in this article.

## Author Contributions

LC designed the project, supervised the research, and drafted and revised the last version of the manuscript. AT conducted the preliminary analyses and wrote the first draft of the manuscript. RM, NH, RSM, and EM conducted the research and data extraction. RM and YW conducted the final data analyses, wrote the parts of the manuscript, and created the figures. TS, YF-W, and SM created the educational intervention, supervised the research implementation, conducted the data collection, and wrote the parts of the manuscript. SM revised the final manuscript. All authors contributed to the article and approved the submitted version.

## Conflict of Interest

The authors declare that the research was conducted in the absence of any commercial or financial relationships that could be construed as a potential conflict of interest.

## Publisher’s Note

All claims expressed in this article are solely those of the authors and do not necessarily represent those of their affiliated organizations, or those of the publisher, the editors and the reviewers. Any product that may be evaluated in this article, or claim that may be made by its manufacturer, is not guaranteed or endorsed by the publisher.
