# Supplementary material for: Educational Intervention for Management of Acute Trauma Pain: A Proof-of-Concept Study in Post-surgical Trauma Patients
Source: Front Psychiatry. 2022 Jul 4;13:853745. doi: 10.3389/fpsyt.2022.853745 (PMC9289147; doi:10.3389/fpsyt.2022.853745)
Supplement: Supplementary file 1 [file Table_1.DOCX]

Instructions: Please fill this out BEFORE looking at the educational materials. **Rate your agreement with the following statements** by drawing a vertical mark on the horizontal line at the point you feel best represents your opinion.

I think all pain related to my trauma is bad and should be treated

Definitely Not Definitely Yes

I think non-medical interventions (music, meditation, ice, elevation, etc.) are an important part of my treatment plan

Definitely Not Definitely Yes

I think Tylenol is an important part of treating my traumatic pain

Definitely Not Definitely Yes

I think Non-steroidal medications (NSAIDs; Advil/Motrin) are an important part of treating my traumatic pain

Definitely Not Definitely Yes

I think opioids/narcotics are an important part of my treatment plan

Definitely Not Definitely Yes

I think I am an important part of the team treating my traumatic pain

Definitely Not Definitely Yes

I expect to be taking narcotics/opioids until I am completely pain free

Definitely Not Definitely Yes

I think a short course of opioids (less than 5 days) is safe

Definitely Not Definitely Yes

What are your goals for your pain management?

________________________________________________________________________________________________________________________________________________

What are the major risks of opioids/narcotics?

________________________________________________________________________________________________________________________________________________

Aside from using opioids/narcotics, are you aware of any other ways to manage pain? Have you tried any of them?

________________________________________________________________________________________________________________________________________________

How have you learned about pain management and opioid use in the past? *(Check all that apply)*

□ Word of Mouth (friends, family, etc.) □ Talking with a doctor

□ Educational materials (brochures, etc.) □ News outlets (online or printed)

□ Social Media □ Advertisements (online or printed) □ Smartphone App

□ Other (please specify) ____________________________________________________

If personalized information about your pain and how to manage it were available to you on a smartphone app, would you use it? □ Yes □ No □ Unsure

Why or why not?

________________________________________________________________________________________________________________________________________________
