# Supplementary material for: Educational Intervention for Management of Acute Trauma Pain: A Proof-of-Concept Study in Post-surgical Trauma Patients
Source: Front Psychiatry. 2022 Jul 4;13:853745. doi: 10.3389/fpsyt.2022.853745 (PMC9289147; doi:10.3389/fpsyt.2022.853745)
Supplement: Supplementary file 2 [file Table_2.DOCX]

**Table 2. Demographic and clinical characteristics**

|  | N/Mean,  95% Confidence Interval  [Lower limit, Upper Limit] |
| --- | --- |
| Sex |  |
| Men | 8 [4,13] |
| Women | 20 [15,24] |
| Age (in years) | 42.04 [35.92,48.15] |
| Marital Status | |
| Married | 9 [5,14] |
| Living as Married | 3 [1,7] |
| Divorced | 4 [1,9] |
| Never Married | 12 [7,17] |
| Ethnicity |  |
| Non-Hispanic | 25 [21,27] |
| Unknown | 3 [1,7] |
| Race |  |
| Black or African American | 15 [10,20] |
| White | 13 [8,18] |
| Education |  |
| Did Not Complete High School | 1 [0,4] |
| Completed High School | 10 [6,15] |
| Some College | 9 [5,14] |
| College Graduate | 3 [1,7] |
| Professional or Post-Graduate Level | 4 [1,8] |
| Not reported | 1 [0,4] |
| Clinical Characteristics | |
| Injury Severity Score | 12.96 [9.85,16.07] |
| Brief Pain Inventory Score | 6.26 [5.62,6.91] |
